# Knowledge Representation and Management: 2023 Highlights and the Rise of Knowledge Graph Embeddings

**DOI:** 10.1055/s-0044-1800748

**Published:** 2025-04-08

**Authors:** Jean Charlet, Licong Cui, Section Editors for the IMIA Yearbook Section on Knowledge Representation and Management

**Affiliations:** 1 Sorbonne Université, INSERM, Univ Sorbonne Paris Nord, LIMICS, Paris, France; 2 AP-HP, DRCI, Paris, France; 3 McWilliams School of Biomedical Informatics, The University of Texas Health Science Center at Houston, Houston, TX, USA

**Keywords:** Knowledge Representation and Management, Knowledge Graph, Ontology, International Medical Informatics Association

## Abstract

**Objectives** : We aim to identify, select, and summarize the best papers published in 2023 for the Knowledge Representation and Management (KRM) section of the International Medical Informatics Association (IMIA) Yearbook.

**Methods** : We performed PubMed queries and adhered to the IMIA Yearbook guidelines for conducting biomedical informatics literature review to select the best papers in KRM published in 2023.

**Results** : Our search yielded a total of 1,666 publications from PubMed. From these, we identified 15 papers as potential candidates for the best papers, and three of them were finally selected as the best papers in the KRM section. The candidate best papers covered three main topics: knowledge graph, knowledge interoperability, and ontology. Notably, two of the three selected best papers explored the potential of knowledge graph embeddings for predicting intensive care unit readmissions and measuring disease distances, respectively.

**Conclusions** : The selection process for the best papers in the KRM section for 2023 showcased a wide spectrum of topics, with knowledge graph embeddings emerging as a promising area for supporting machine learning applications in biomedicine.

## 1. Introduction

The field of Knowledge Representation and Management (KRM) in medicine experienced a prolific year in 2023, with numerous publications emerging. KRM plays a crucial role by providing essential resources and methods for various medical informatics domains [ 1 2 3 4 5 6 7 ]. This synopsis details the selection process employed to identify the best papers for the KRM section of the 2024 International Medical Informatics Association (IMIA) Yearbook. We also provide a summary of the key findings from the nominated candidate best papers.

## 2. Paper Selection Method

We conducted a literature search based on PubMed/MEDLINE to identify papers related to KRM in the field of medical informatics. This search focused on articles published in peer-reviewed international journals and conference proceedings indexed by PubMed. We utilized the same query set from last year with Medical Subject Headings (MeSH) descriptors [ 7 ]. Our inclusion criteria were original research articles published between January 1, 2023, and December 31, 2023, while excluding publication types such as reviews, editorials, comments, case reports, and letters to editors.

We followed the usual process [ 8 ] typically employed by the IMIA Yearbook sections to select the best papers. This process comprised three stages. Initially, the section editors screened papers based on their titles, abstracts, and publication types. Following this, a second review was conducted to shortlist a set of candidate best papers for peer review. Each short-listed paper was then reviewed and assessed by the two KRM section editors, one IMIA chief editor, and two external reviewers. The evaluation criteria encompassed topic importance, scientific and practical impact, scientific content, originality, quality of literature, and quality of presentation. The final selection of the best papers was made during a meeting of the entire editorial board, taking into account the peer review results and the section editors' reports.

## 3. Results

### 3.1 Best Paper Selection for 2023

We retrieved a total of 1,666 KRM-related papers published in 2023 from PubMed. After the section editors' initial screening, we obtained 222 papers. The section editors further reviewed these papers jointly and reached a consensus list of 15 papers, which were nominated as the candidate best papers [ 9 10 11 12 13 14 15 16 17 18 19 20 21 22 23 ]. External reviewers, IMIA Yearbook editors and section editors further evaluated these 15 papers and finally selected three best papers (see Table 1 ).

**Table 1.** Best paper selection of articles for the IMIA Yearbook of Medical Informatics 2024 in the ‘Knowledge Representation and Management’ section. The articles are listed in alphabetical order of the first author's surname.

Section Knowledge Representation and Management
Carvalho RM, Oliveira D, Pesquita C. Knowledge Graph Embeddings for ICU readmission prediction. BMC Med Inform Decis Mak 2023;23:12.
Caufield JH, Putman T, Schaper K, Unni DR, Hegde H, Callahan TJ, Cappelletti L, Moxon SAT, Ravanmehr V, Carbon S, Chan LE, Cortes K, Shefchek KA, Elsarboukh G, Balhoff J, Fontana T, Matentzoglu N, Bruskiewich RM, Thessen AE, Harris NL, Munoz-Torres MC, Haendel MA, Robinson PN, Joachimiak MP, Mungall CJ, Reese JT. KG-Hub-building and exchanging biological knowledge graphs. Bioinformatics 2023;39(7):btad418.
Fu M, Yan Y, Loohuis LM, Chang TS. Defining the distance between diseases using SNOMED CT embeddings. J Biomed Inform 2023;139:104307.

The first article is a contribution by Carvalho et al. [ 9 ], where the authors developed an approach to predicting Intensive Care Unit (ICU) readmissions by integrating semantic annotations and Knowledge Graphs (KGs) into Electronic Health Record (EHR) data. More specifically, the authors enriched EHR data with semantic annotations to ontologies and constructed a KG capturing the relationships between data entities. This enriched data was then used to develop machine learning models to predict 30-day ICU readmissions. These models leveraging KG embeddings outperformed other existing methods. This study showcased the potential benefits of integrating ontologies and KGs into biomedical machine learning applications.

In the second paper, Caufield et al. [ 10 ] developed KG-Hub, a platform specifically designed to address the challenges of constructing, exchanging, and reusing biomedical KGs. KG-Hub offers a standardized approach for building KGs that are compatible with the Biolink model and integrates seamlessly with Open Biological and Biomedical Ontologies (OBO). Additionally, it facilitates the sharing and reuse of KGs between projects, supporting a range of applications such as COVID-19 research and rare disease studies. KG-Hub even integrates with graph machine learning tools allowing researchers to analyze and exploit KGs for further discovery.

The third article, authored by Fu et al. [ 11 ], introduced a new distance metric based on the KG embeddings of SNOMED CT to define disease distances for all disease pairs in ICD-10 (International Classification of Diseases, version 10). When compared to three other distance metrics derived from the hierarchical structure of ICD-10, clinical comorbidity, and genetic correlation, the KG embedding-based metric demonstrated a higher level of granularity in capturing known phenotypic, clinical, and molecular characteristics.

Further content summaries of the three best papers are provided in the appendix of this synopsis.

The 15 candidate best papers fall into three major categories: KG, knowledge interoperability, and ontology. Particularly noteworthy is the potential of KG embeddings to support machine learning applications in biomedicine.

### 3.2 Knowledge Graph

Six articles [ 9 10 11 12 13 14 ] fall under the category of KG, including the three best papers [ 9 10 11 ]. Among these, three articles are centred on KG embeddings [ 9 , 11 , 12 ], two are related to the KG development and applications [ 13 , 14 ], and one is with regard to the tools for KG development and management (i.e., KG-Hub mentioned above [ 10 ]).

Besides the two best papers [ 9 ] and [ 11 ] that investigated KG embeddings for ICU readmission prediction and defining disease distance, the candidate paper [ 12 ] by Nunes et al. utilized KG embeddings to predict gene-disease associations. The authors leveraged rich semantic representations derived from KG embeddings across multiple ontologies (Human Phenotype Ontology and Gene Ontology). They employed ontology logical definitions and ontology mappings to explore different strategies for constructing semantic representations of genes and diseases, and assessed the performance impact of these strategies on various KG embedding techniques. This study showcased the potential of using KG embeddings from multiple linked ontologies to facilitate gene-disease prediction.

The candidate paper [ 13 ] by Taneja et al. presents the development of NP-KG, a KG designed to investigate pharmacokinetic interactions between natural products and pharmaceutical drugs. NP-KG integrates data from biomedical ontologies, drug databases, and scientific literature. The evaluation of the NP-KG with green tea and kratom as use cases showed that it contains both congruent and contradictory information compared to curated data, highlighting the importance of considering multiple sources of evidence. Overall, NP-KG provides a valuable resource for studying and predicting natural product-drug interactions.

Abad-Navarro et al. [ 14 ] developed a KG-based data harmonization framework designed to facilitate secondary reuse of healthcare data across institutions. The framework features an ontology-based common data model, a data transformation pipeline, and a semantic query system, enabling the integration and semantic interoperability of heterogeneous data. Implementation of this framework in the European H2020 Precise4Q project demonstrated its capability to semantically integrate and query diverse healthcare data, thereby enhancing the potential for advanced data analysis and machine learning model development.

### 3.3 Knowledge Interoperability

In the candidate paper [ 15 ], Lario et al. introduced a reference knowledge framework called “Multilayer Metamodel for Representation and Knowledge” (M*R/K) to address the challenges of representing and sharing medical knowledge in clinical information systems. By incorporating frameworks from multiple disciplines such as healthcare, linguistics, and system engineering, M*R/K establishes a structured approach for developing and managing medical knowledge artifacts such as clinical practice guidelines. M*R/K provides a standardized vocabulary and systematic method for organizing and articulating medical knowledge curation perspectives, concepts, and relationships, facilitating better capture, sharing, and implementation in clinical information systems like EHRs.

In another candidate paper [ 16 ], McDonald et al. investigated the accuracy of mapping local laboratory test codes in two PCORnet DataMarts to the Logical Observation Identifiers Names and Codes (LOINC), which is critical for data integration across healthcare systems. They examined 179 million test results using software tools and manual review, and found a 4.6% overall mapping error rate. The authors also proposed an algorithm for automatically detecting and correcting mapping errors, which reduced the error rate to 0.1%. Additionally, this study suggested making LOINC codes available to clinical users and administrators in EHR systems to identify and correct mapping inconsistencies for their data integration needs.

### 3.4 Ontology

Similar to the previous year [ 7 ], ontology research still attracts considerable attention. Out of the 15 candidate papers, six focus on ontology development and/or applications, and one is concerned with ontology evolution.

Raboudi et al. [ 17 ] developed the PACIFIC ontology, a sophisticated ontology to manage multimodal and multi-source data for heart failure with preserved ejection fraction (HFpEF). The ontology extends the Biomedical Study-Lifecycle Management (BMS-LM) core ontology and incorporates concepts from Medical Subject Headings (MeSH) and Uberon anatomy ontology. It also includes annotations with referencing codes from other standards such as LOINC. By organizing 1,372 variables with extensive annotations, the PACIFIC ontology provides a robust cardiac data management framework for patient phenomapping and enables the interoperability of multimodal cardiac datasets.

The candidate paper [ 18 ] by Zhang et al. presents the development and application of the integrated Alzheimer's Disease Ontology (ADO). By incorporating concepts from the previous versions of ADO and the Alzheimer's Disease Mapping Ontology (ADMO) and integrating new findings on risk factor genes and treatments, the ADO provides a comprehensive framework of 39,855 axioms covering various critical aspects of Alzheimer's disease (AD). The paper also showcases the ADO's role in text mining applications, particularly in extracting data from the SCAIView database, demonstrating its value in advancing AD research.

Taglino et al. [ 19 ] developed a computational ontology called AD-Onto focusing on the domain of the neuropsychological tests, inspired by the Alzheimer's Disease Neuroimaging Initiative (ADNI) data collection. They also created a software tool, OntoLoader, for populating the ontology with ADNI data and enabling complex queries. The proposed ontology improves access to the ADNI dataset, facilitates intuitive querying, and allows for the application of machine learning techniques. This work has implications for the design and implementation of information systems for AD data collection and management, as well as for the harmonization and integration of AD data from different sources.

The paper from Hernández et al. [ 20 ] focuses on creating HeNeCOn, the first ontology tailored for Head and Neck Cancer (HNC), to standardize the diverse clinical data associated with this disease. HeNeCOn integrated 170 clinical variables into a taxonomy, formalizing and organizing them into 502 classes with semantic definitions of 283 medical terms. The iterative development process involved extensive review and validation by clinicians and statisticians. HeNeCOn can be used to organize and standardize clinical data related to HNC prognosis, treatment, and follow-up, facilitating information extraction, knowledge management, and data integration in HNC research.

Dong et al. [ 21 ] explored computational text phenotyping to identify rare diseases from clinical notes using an ontology-driven framework and weak supervision. By leveraging pre-trained contextual representations like BERT, this method includes two key steps: extracting phenotypes by linking text to the Unified Medical Language System (UMLS) concepts, and matching these concepts to rare diseases in the Orphanet Rare Disease Ontology. Evaluations on datasets from MIMIC-III and NHS Tayside showed significant improvements in precision for Text-to-UMLS linking without sacrificing recall. The findings suggest that this weakly supervised Natural Language Processing (NLP) approach can enhance rare disease identification in clinical notes, offering a complementary way to the traditional ICD-based approach.

The paper from Calvo-Cidoncha et al. [ 22 ] presents OntoPharma, an ontology-based clinical decision support system, designed to improve medication appropriateness in older patients with multimorbidity. The study focuses on modeling the chronic patient domain and addresses medication regimen complexity, anticholinergic and sedative drug burden, and triggers for adverse events. OntoPharma generates alerts based on these factors and has been implemented in a hospital setting, demonstrating its feasibility and effectiveness in clinical practice.

Diaz Benavides et al.'s work from the candidate paper [ 23 ] is related to ontology evolution. This work focuses on the development and evaluation of DynDiff, a tool for comparing and analyzing changes in large and dynamic ontologies. The tool computes and categorizes changes into basic, complex, and heuristic types, providing a detailed description of the changes. This work also introduces DynDiffOnto, an ontology that defines a set of change actions to support the interpretation of ontology evolution. The evaluation of DynDiff demonstrates its efficiency, scalability, and ability to identify high-level changes, making it a valuable tool for understanding the evolution of ontologies.

## 4. Conclusions

The selected 15 candidate best papers for the KRM section in the year 2023 fell into three major categories: KG, knowledge interoperability, and ontology. The KG papers focused on KG embeddings, KG development and applications, and tools for KG development and management. The papers in the knowledge interoperability category addressed the challenges of representing and sharing medical knowledge in clinical information systems and mapping local laboratory test codes to standard codes. The ontology papers covered the development and applications of domain ontologies, such as for Alzheimer's Disease and Head and Neck Cancer, as well as ontology evolution.

The continued attention on ontology research underscores its essential role in structuring and standardizing medical knowledge. The development of specialized ontologies for various medical domains, along with tools for ontology evolution analysis, demonstrates ongoing efforts to enhance data interoperability, knowledge management, and clinical decision support systems. Notably, KG embeddings have emerged as a promising area for supporting machine learning applications in biomedicine.

Overall, the papers identified in the KRM section showcased the importance of KRM in advancing biomedical informatics and supporting various applications in biomedicine.
